# Assessment of Clinicopathological Response to Neoadjuvant Chemotherapy in Patients Diagnosed With Locally Advanced Breast Carcinoma

**DOI:** 10.7759/cureus.82057

**Published:** 2025-04-11

**Authors:** Avegu Balji, Radhakrishna Ramchandani, Sarita Ramchandani, Amit Chowhan, Amit Agarwal, Yashwant Kashyap

**Affiliations:** 1 General Surgery, All India Institute of Medical Sciences, Raipur, Raipur, IND; 2 Anesthesiology, All India Institute of Medical Sciences, Raipur, Raipur, IND; 3 Pathology and Laboratory Medicine, All India Institute of Medical Sciences, Raipur, Raipur, IND; 4 Medical Oncology, All India Institute of Medical Sciences, Raipur, Raipur, IND; 5 Medical Oncology, Balco Medical Centre, Naya Raipur, IND

**Keywords:** clinical response, locally advanced breast cancer, neoadjuvant chemotherapy, pathological response, patient assessment

## Abstract

Introduction

Globally, breast carcinoma is the most prevalent carcinoma and the primary cause of cancer-related deaths in women. Neoadjuvant chemotherapy (NACT) is a widely used treatment approach for patients with locally advanced breast carcinoma (LABC), as it reduces the size of the tumor, making previously inoperable tumors operable. Studies suggest that patients with LABC achieve a high response rate following NACT. Still, no such research has been done to date in central India. So, this study was designed to evaluate the clinical and pathological response to NACT in patients diagnosed with LABC who presented at a tertiary care institute in central India. The primary outcome of our study was the clinical and pathological response following NACT in patients diagnosed with LABC. The secondary outcome was the comparison of the response to NACT in different molecular receptor subtypes of LABC.

Methods

After approval from the IEC (2044/IEC-AIIMSRPR/2021, dated 30/11/2021) and written informed consent from the patients, this study was carried out at All India Institute of Medical Sciences, Raipur, from January 2022 to January 2023. Fifty-six women, aged 18 years and above, diagnosed with LABC, were included in this study. Patients who were lost to follow-up, did not give consent, underwent previous cancer treatment in any form, had unknown immunohistochemistry (IHC) status, had inconclusive pathological reports, had fungating/ulcerative breast lesions, were pregnant or lactating, and desired to be pregnant shortly were excluded. Demographic details of the patients and baseline tumor characteristics were noted. Patients diagnosed with LABC were administered NACT using a standard regimen. After three months, the clinical and pathological response of the tumor to NACT was assessed. Patients who responded to chemotherapy underwent surgery, while those with static or progressive disease were continued on chemotherapy for an additional three cycles and then reassessed. Based on the histopathological report of the postoperative specimen, the subtype and grading of the tumor were done, and an assessment of the clinical and pathological response of the tumor to NACT in various subtypes was made.

Results

Fifty-six patients receiving NACT for LABC were studied. Post-NACT, significant improvement in tumor characteristics, including size, hardness, and fixity to the skin or muscle, was noted (P<0.05), and the number of patients with axillary and supraclavicular lymphadenopathy decreased significantly (P<0.05). A significant rise in the number of patients belonging to T0-T2 and N0-N1 stages, along with a decline in the number of participants belonging to T3-T4 and N2-N3 stages, was noted, suggesting a remarkable downstaging of the tumor (P=0.001) post-NACT. A remarkable improvement in the grading of the tumor was noted (P=0.001). Out of 56 patients, in 12 (21.4%) patients, a complete pathological response (cPR) of the tumor was noted, while in the remaining 44 patients, there was no change in the ER/PR and HER2-neu receptor status. Triple-negative breast cancer (TNBC) had a maximum cPR rate of 12.5%.

Conclusion

Post-NACT, a significant downstaging and downgrading of the tumor and downstaging of lymph nodes were noted. We conclude that NACT has a definitive role in patients diagnosed with LABC as far as the clinical and pathological response of the tumor is concerned. Also, the TNBC subtype responds maximally to NACT and carries a better prognosis.

## Introduction

Globally, breast carcinoma is not only the most commonly occurring and most prevalent carcinoma in women but also the primary cause of carcinoma-related deaths in women [1,2]. Among various therapeutic modalities, modified radical mastectomy (MRM) remains the most commonly done surgical treatment for breast carcinoma. The goal of mastectomy is to remove breast tissue containing the tumor, along with the ductal and lobular structures. If this is not achieved, residual tumor cells from the primary focus may lead to tumor recurrence. Due to this reason, there is locoregional recurrence and reduced long-term survival [3].

Neoadjuvant chemotherapy (NACT) is a commonly used approach for patients with locally advanced breast carcinoma (LABC). It often reduces the size of the tumor significantly, making previously inoperable tumors suitable for potentially curative surgery [4]. Following NACT, around 30% of patients with LABC achieve either a partial or complete response, thus improving overall survival. The early administration of NACT can help limit micrometastasis and control the disease [5]. The prognosis is better in patients diagnosed with early breast disease and a smaller number of axillary lymph nodes. Survival is poor in patients having a larger breast tumor with a higher number of axillary lymph nodes [6,7].

The main objective of NACT is to decrease the tumor size and burden of axillary lymph nodes, achieving either a partial or complete pathological response (cPR) and enhancing relapse-free survival. Several studies suggest that patients with LABC achieve a high response rate after NACT with a full pathological and clinical response to primary breast carcinoma. Still, no such research has been done to date in central India, so this study was designed to evaluate the clinical and pathological response of the tumor to NACT in patients diagnosed with LABC and reporting to a tertiary care institute in central India.

The primary outcome of our study was the clinical and pathological response of the tumor to NACT in patients diagnosed with LABC and reporting to a tertiary care institute in central India from January 2022 to January 2023. The secondary outcome was the comparison of the response to NACT in different molecular receptor subtypes of LABC.

## Materials and methods

The procedures followed in this study were per the standards of the "Institute Ethics Committee" (IEC) and the "Declaration of Helsinki (2013)." With due approval from the IEC (2044/IEC-AIIMSRPR/2021, dated 30/11/2021) and written informed consent from the participants, the study was executed from January 2022 to January 2023 at a tertiary care center situated in central India in the Chhattisgarh state named All India Institute of Medical Sciences (AIIMS), Raipur.

Fifty-six female patients, aged 18 years and above, diagnosed with LABC, were included in this study. Patients who were lost follow-up; did not give consent; underwent previous cancer treatment in any form such as radiotherapy, chemotherapy, or surgery; had unknown immunohistochemistry (IHC) status, an inconclusive pathological report, a fungating and/or ulcerative breast lesion; were pregnant or lactating; and were desirous to be pregnant either in the study period or the near future were excluded. Demographic details of the participants and baseline tumor characteristics such as stage/grade, status of lymph nodes, estrogen receptor (ER), progesterone receptor (PR), and human epidermal growth factor receptor 2 (HER2)-neu receptor were recorded. Tumor size and nodal status were evaluated through clinical examination and sono-mammography. A tru-cut biopsy was taken from all the participants, and IHC with or without fluorescent in situ hybridization (FISH) was done for the analysis of the tumor tissue for receptor status. ER/PR status positivity was defined as nuclear staining in more than 1% of tumor cells, and HER2 positivity was determined by a 3+ score on IHC or a FISH ratio exceeding 2.0.

By definition, LABC includes any T from T0-T4 and any N from N0-N3 without distant metastasis, excluding early breast cancer [8]. According to the tumor-node-metastasis (TNM) staging system, LABC is classified as stage II B (T2-N1; T3-N0), III A (T0/1/2-N2; T3-N1/2), III B (T4; N0-2), and III C (any T; N3) [9]. Patients with a confirmed diagnosis of LABC were subjected to NACT using the standard regimen of four cycles of anthracycline (epirubicin 90 mg/m^2^ or Adriamycin 60 mg/m^2^), along with cyclophosphamide (600 mg/m^2^) and four cycles of paclitaxel (175 mg/m^2^). Patients having HER2-neu receptor positive status received an injection (Inj) of trastuzumab, 8 mg/kg for the first cycle and 6 mg/kg from the second cycle onward, along with paclitaxel. In the case of locally advanced triple-negative breast cancers (TNBC), carboplatin at a dose of the area under the curve-five (AUC-5) was added to paclitaxel. All chemotherapy regimens were given at an interval of three weeks. After administering NACT for three months, the clinical and pathological assessment of the tumor was done. Clinical assessment was done to look for the regression of the tumor, involvement of surrounding structures, and status of axillary and draining lymph nodes. The tumor was assessed pathologically as per the Bloom-Richardson-Elston system, also called the Nottingham system for breast cancer grading, where each feature had a score ranging from 1 to 3 (Table 1) [10].

**Table 1:** Bloom-Richardson-Elston system for the grading of breast cancer A cumulative score ranging from 3 to 5 indicates a well-differentiated grade 1 tumor, 6 to 7 corresponds to a moderately differentiated grade 2 tumor, and 8 to 9 indicates a poorly differentiated grade 3 tumor *The use of field dimensions of 0.27 square millimeters HPFs: high-power fields

Features	Description	Score
Gland and tubule formation	A majority of tumors show well-formed glands and tubules (>75%)	1
	A moderate degree of gland and tubule formation (10%-75%)	2
	Little or no gland/tubule formation (<10%)	3
Nuclear pleomorphism	Small, regular uniform cells	1
	A moderate rise in size and variability	2
	Marked variation	3
Mitotic count per 10 HPFs*	0-9	1
	10-19	2
	>20	3

Response Evaluation Criteria in Solid Tumors (RECIST) 1.1 was used to assess the response of the tumor post-NACT [11]. Patients who responded to chemotherapy underwent surgery, while those with static or progressive disease were continued on chemotherapy for an additional three cycles and were then reassessed. Based on the histopathological examination (HPE) report of the postoperative specimen, the subtype and grading of the tumor were determined. On the basis of molecular subtypes, carcinoma can be classified as a luminal A subtype being hormone-positive while HER2-neu-negative, a luminal B subtype being both hormone- and HER2-neu-positive, a TNBC subtype being both hormone- and HER2-neu-negative, and a HER2-neu-enriched subtype being hormone-negative while HER2-neu-positive. Post-surgery, the assessment and comparison of the clinical and pathological response of the tumor to NACT in these subtypes were done. Patients were considered to have a complete pathological response (cPR) if, post-NACT, there were no residual invasive carcinoma cells either in breast tissue or in the axillary lymph nodes. This also included cases with residual ductal carcinoma in situ (DCIS) without invasive disease.

The sample size was determined based on the findings of the study done by Kunnuru et al. in 2020, in which the authors noted N2 stage disease in 20% of patients before chemotherapy, which reduced to 6.7% post-chemotherapy [12]. The following formula was used for sample size estimation: N=(p*)(1-p*)(Zβ+Zα/2)²/(p1-p2)², where p1 and p2 are the percentage of patients with N2 stage disease pre- and post-chemotherapy, respectively; p* is the average of proportions; Zα/2=1.96 at a 95% confidence interval; and Zβ=0.84 at 80% power.

Substituting the values, a sample size of approximately 51 was derived. Considering an additional 10% dropout, a final sample size of 56 was taken up.

The data were recorded in a Microsoft (MS) Excel spreadsheet version 365 (Microsoft Corporation, Redmond, WA), while IBM SPSS 21 (IBM Corp., Armonk, NY) for Windows was used for statistical analysis. Categorical data were expressed as numbers/percentages. Continuous data were represented by mean±standard deviation (SD) or median and interquartile range (IQR). Data were presented in a graphical manner wherever appropriate for better visualization. The Wilcoxon signed-rank test was used for the paired statistical comparison of the categorical variables. Statistical significance was set at a P-value of less than 0.05.

## Results

**Figure 1:**
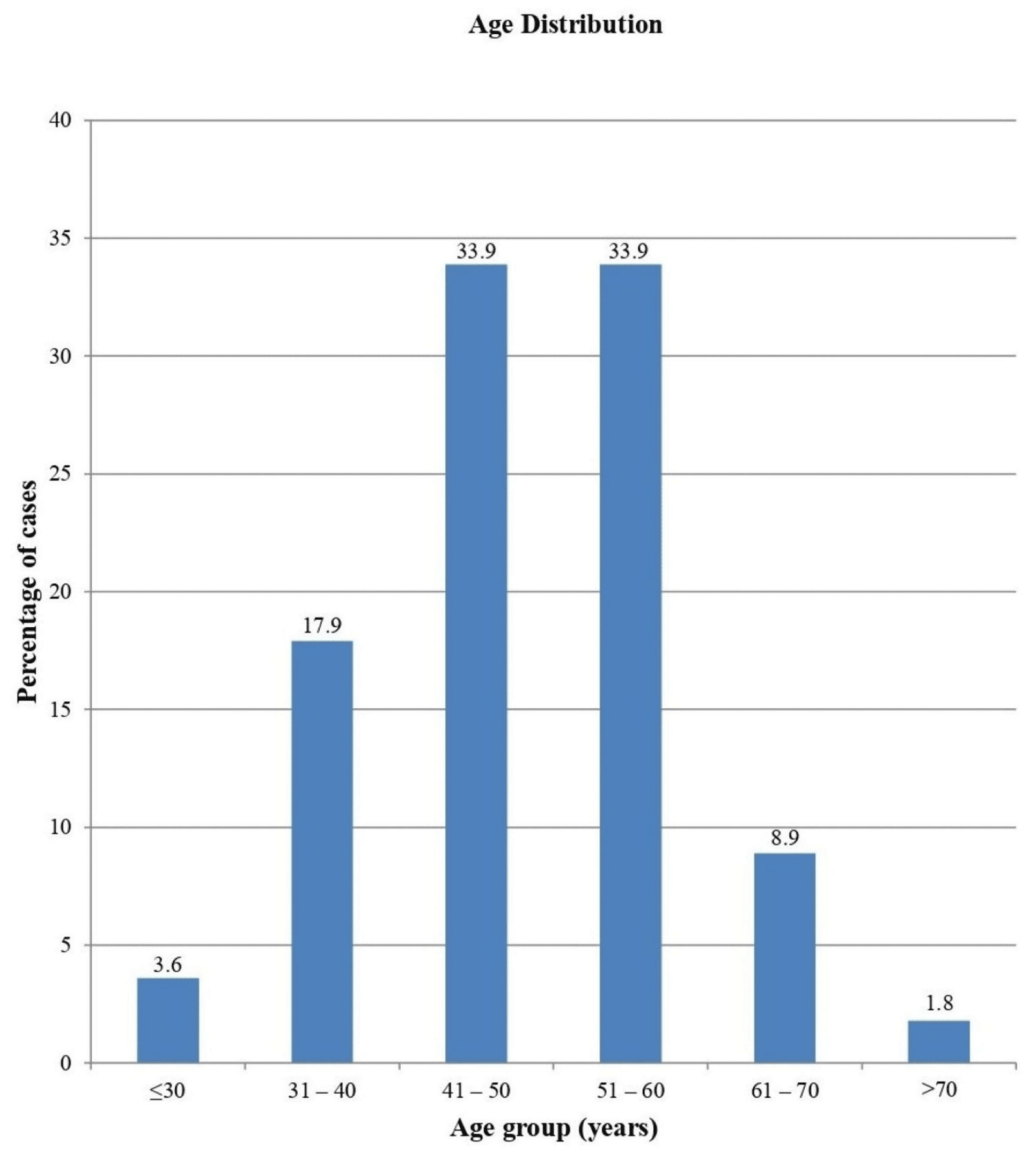
Age distribution

In our study, 56 female patients receiving NACT for LABC were studied. A maximum number of the participants (around 68%) belonged to the age group of 41-60 years (Figure 1). The median (IQR) of the age of the participants was 49.42 (13.79) years. The minimum and maximum ages varied from 28 to 72 years, respectively.

**Figure 2:**
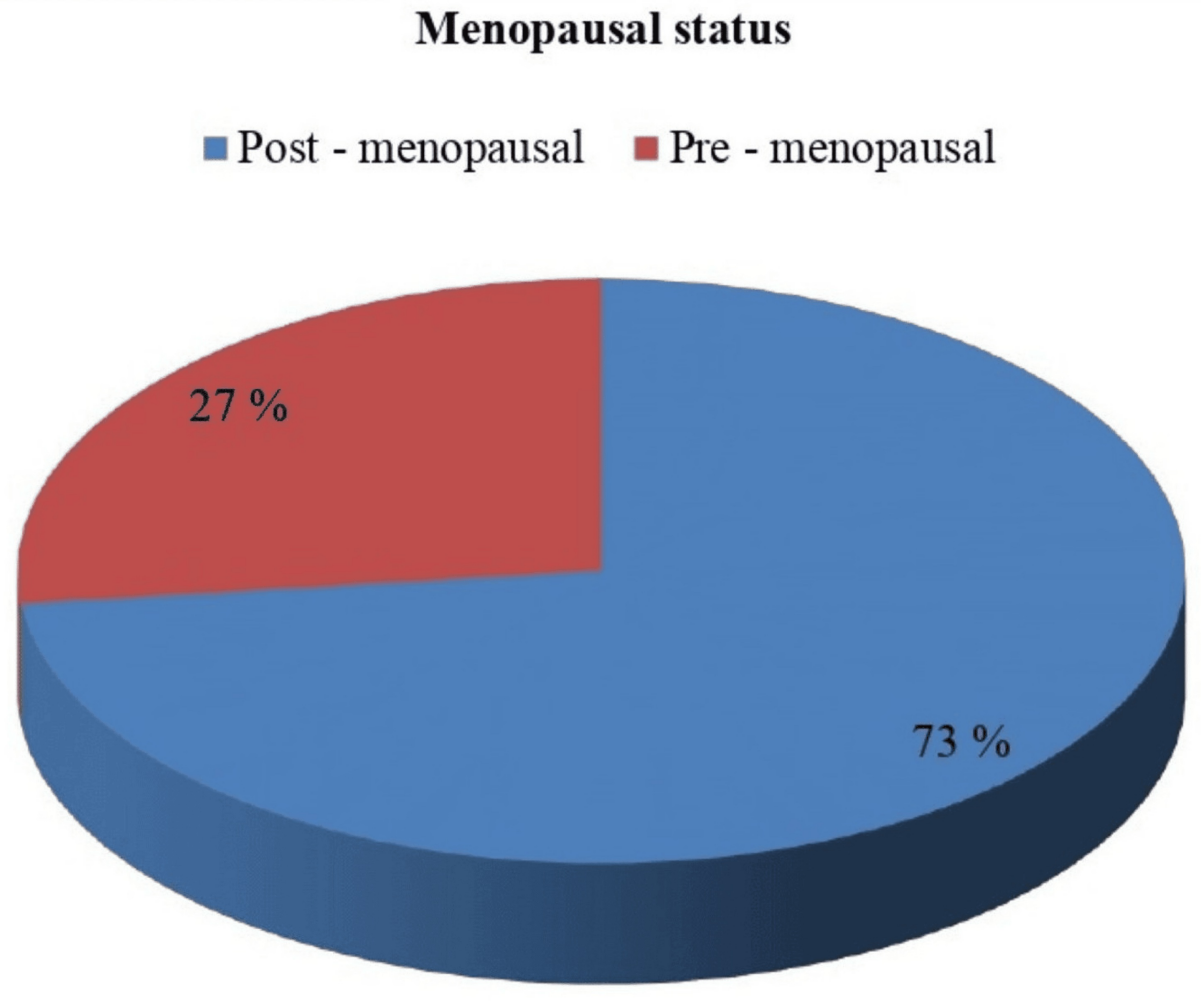
Menopausal status

As far as menopausal status was concerned, 15 (27%) patients belonged to the pre-menopausal group, whereas 41 (73%) patients belonged to the post-menopausal group (Figure 2).

Tumor characteristics were assessed pre- and post-NACT in all the patients. Regarding the size of the tumor, a notable rise in the number of patients with tumors smaller than 5 cm was noted from 22 (39.2%) patients pre-NACT to 52 (92.9%) patients post-NACT. Likewise, a remarkable fall in the number of patients with tumors measuring 5-8 cm in size was noted from 27 (49.1%) cases pre-NACT to four (7.3%) cases post-NACT. Seven (12.7%) patients had tumors larger than 8 cm pre-NACT, whereas none of the patients had tumors larger than 8 cm post-NACT. The number of patients with tumors classified as "hard" dropped significantly from 44 (78.6%) cases pre-NACT to 18 (32.1%) cases post-NACT. Similarly, the number of patients with tumors classified as "not hard" increased significantly from 12 (21.4%) cases pre-NACT to 38 (67.9%) cases post-NACT (P=0.001). As far as the fixation of the tumor to the skin or muscle was concerned, a significant reduction in the number of patients having the tumor fixed to the skin or muscle was noted from 13 (23.2%) cases pre-NACT to eight (14.3%) cases post-NACT (P=0.025). Similarly, the number of patients having tumors without fixation to the skin or muscle increased from 43 (76.8%) cases pre-NACT to 48 (85.7%) cases post-NACT (P=0.025). These results demonstrate a statistically significant improvement in tumor characteristics following NACT (Table 2).

**Table 2:** Pre- and post-NACT tumor characteristics Data are presented as numbers (percentages) ^1^Wilcoxon signed-rank test *A statistically significant P-value of <0.05 n, number; %, percentage; cm, centimeters; NACT, neoadjuvant chemotherapy

Tumor characteristics		Pre-NACT (n=56)		Post-NACT (n=56)		P-value^1^
		n	%	n	%	
Tumor size (cm)	<5	22	39.2	52	92.9	0.001^*^
	5-8	27	49.1	4	7.3	
	>8	7	12.7	0	0.0	
Hardness	Yes	44	78.6	18	32.1	0.001^*^
	No	12	21.4	38	67.9	
Fixation to the skin/muscle	Yes	13	23.2	8	14.3	0.025^*^
	No	43	76.8	48	85.7	

The lymph node status of all the patients was assessed pre- and post-NACT. The number of patients with axillary lymphadenopathy decreased significantly from 46 (82.1%) patients pre-NACT to 10 (17.9%) patients post-NACT, and the number of patients without axillary lymphadenopathy increased from 10 (17.9%) patients pre-NACT to 46 (82.1%) patients post-NACT (P=0.001). Similarly, the number of patients with supraclavicular lymphadenopathy decreased from 10 (17.9%) patients pre-NACT to two (3.6%) patients post-NACT, and the number of patients without supraclavicular lymphadenopathy increased from 46 (82.1%) cases pre-NACT to 54 (96.4%) cases post-NACT (P=0.005). The status of the infraclavicular lymph nodes remained unchanged (P=1.000) pre- and post-NACT (Table 3).

**Table 3:** Pre- and post-NACT lymph node status Data are presented as numbers (percentages) ^1^Wilcoxon signed-rank test *A statistically significant P-value of <0.05 n, number; %, percentage; NACT, neoadjuvant chemotherapy

Lymphadenopathy	Result	Pre-NACT (n=56)		Post-NACT (n=56)		P-value^1^
		n	%	n	%	
Axillary	Yes	46	82.1	10	17.9	0.001*
	No	10	17.9	46	82.1	
Supraclavicular	Yes	10	17.9	2	3.6	0.005*
	No	46	82.1	54	96.4	
Infraclavicular	Yes	1	1.8	1	1.8	1.000
	No	55	98.2	55	98.2	

As per the TNM staging, a notable rise in the number of patients belonging to the T0-T2 stage was noted from 22 (39.3%) patients pre-NACT to 52 (92.9%) patients post-NACT. Likewise, a notable decline in the number of patients having T3-T4 stage disease was noted from 34 (60.7%) to four (7.3%) pre-NACT versus post-NACT (P=0.001). A notable rise in the number of participants belonging to the N0-N1 stage was noted from 33 (58.9%) to 51 (91.1%) patients pre-NACT versus post-NACT, along with a remarkable fall in the number of patients belonging to the N2-N3 stage from 23 (41.1%) to five (8.9%) patients pre-NACT versus post-NACT (P=0.001) (Table 4).

**Table 4:** Pre- and post-NACT tumor and nodal staging Data are presented as numbers (percentages) ^1^Wilcoxon signed-rank test *A statistically significant P-value of <0.05 n, number; %, percentage; T, tumor; N, nodal; NACT, neoadjuvant chemotherapy

Parameters	Staging	Pre-NACT (n=56)		Post-NACT (n=56)		P-value^1^
		n	%	n	%	
T	T0-T2	22	39.3	52	92.9	0.001^*^
	T3-T4	34	60.7	4	7.3	
N	N0-N1	33	58.9	51	91.1	0.001^*^
	N2-N3	23	41.1	5	8.9	

As per the histological classification, out of 56 patients, 12 (21.4%) patients had normal HPE findings post-NACT due to a cPR of the tumor. As per tumor classification, a significant reduction in the number of patients having infiltrating ductal carcinoma (IDC) was noted from 55 (98.2%) patients pre-NACT to 43 (76.8%) patients post-NACT. However, there was no change in the number of patients having infiltrating lobular carcinoma (ILC) pre- and post-NACT. Thus, it can be inferred that IDC responded well to NACT (P=0.001). As far as tumor grading is concerned, only one (1.8%) patient had a grade 1 tumor pre-NACT as compared to three (5.4%) patients post-NACT. Similarly, 43 (76.8%) patients had grade 2 tumors pre-NACT, which decreased to 35 (62.5%) patients post-NACT. Likewise, 12 (21.4%) patients had grade 3 tumors pre-NACT, which decreased to six (10.7%) patients post-NACT. Thus, a remarkable improvement in the grading of the tumor was noted post-NACT compared to the pre-NACT period, with a P-value of 0.001 (Table 5).

**Table 5:** Classification and grading of tumor pre- and post-NACT based on HPE findings Data are presented as numbers (percentages) ^1^Wilcoxon signed-rank test *A statistically significant P-value of <0.05 NA, not applicable; n, number; %, percentage; HPE, histopathological examination; NACT, neoadjuvant chemotherapy

HPE findings	Pre-NACT (n=56)		Post-NACT (n=56)		P-value^1^
	n	%	n	%	
Tumor classification					
Infiltrating ductal carcinoma	55	98.2	43	76.8	0.001^*^
Infiltrating lobular carcinoma	1	1.8	1	1.8	1.000
None	NA	NA	12	21.4	-
Grade of tumor					
Grade 1	1	1.8	3	5.4	0.001^*^
Grade 2	43	76.8	35	62.5	0.001^*^
Grade 3	12	21.4	6	10.7	0.001^*^
No residual tumor found	NA	NA	12	21.4	-

In our study, in 44 out of 56 patients, no change was found in the ER, PR, and HER2-neu receptor status pre-NACT versus post-NACT. In the remaining 12 patients, the information regarding the transformation of receptor status post-NACT could not be evaluated due to the cPR of the tumor following NACT. As per the classification based on molecular subtypes, it was found that out of 56 patients, three patients belonged to luminal A, 21 patients belonged to luminal B, 15 patients belonged to HER2-neu-positive, and 17 patients belonged to TNBC subtype pre-NACT. Among these, a cPR was noted in 12 patients post-NACT, comprising two patients belonging to HER2-neu-positive, three patients belonging to luminal B, and seven patients belonging to the TNBC subtype. The number of patients belonging to the luminal A subtype remained the same. Thus, it is inferred that post-NACT, TNBC had a maximum cPR rate of 12.5% (Table 6).

**Table 6:** Molecular subtypes pre- and post-NACT *Data could not be retrieved in 12 patients due to the complete pathological response of the tumor post-neoadjuvant chemotherapy TNBC, triple-negative breast cancer; NACT, neoadjuvant chemotherapy; HER2, human epidermal growth factor receptor 2

Molecular subtypes	Pre-NACT (n=56)		Post-NACT* (n=56)	
	n	%	n	%
Luminal A	3	5.4	3	5.4
Luminal B	21	37.5	18	32.1
HER2-neu-positive	15	26.7	13	23.2
TNBC	17	30.4	10	17.9
Not available*	0	0.0	12	21.4
Total	56	100.0	56	100.0

## Discussion

In our study, a maximum number of patients (around 68%) were in the age group of 41-60 years. The median (IQR) of age was 49.42 (13.79) years. The minimum and maximum ages of the patients varied from 28 to 72 years. As far as the menopausal state is concerned, 15 (27%) patients belonged to the pre-menopausal state, while 41 (73%) patients belonged to the post-menopausal state. The reason behind this finding could be that breast carcinoma is rarely encountered in children and adolescents, and thereafter, incidence increases with age, which reaches its peak at 50-60 years of age. The age-associated increased prevalence is related to prolonged exposure to estrogen hormones, which can be because of early menarche, late menopause, advanced age at the time of first childbirth, the consumption of oral contraceptive pills, and hormone replacement therapy [13].

On the assessment of tumor characteristics, a statistically significant rise in the number of patients having tumors smaller than 5 cm was noted post-NACT as compared to pre-NACT. Similarly, a statistically significant decline in the number of patients with tumors measuring 5-8 cm in size was noted post-NACT as compared to pre-NACT. Post-NACT, none of the patients had tumors larger than 8 cm. Thus, a significant decrease in the size of the tumors was noted post-NACT in our study. As far as TNM staging was concerned, a significant rise in the number of participants belonging to the T0-T2 and N0-N1 stages was noted, along with a remarkable decline in the number of participants belonging to the T3-T4 and N2-N3 stages after receiving NACT as compared to the pre-NACT period, revealing a remarkable downstage. The number of patients with axillary lymphadenopathy decreased significantly from 46 (82.1%) pre-NACT to 10 (17.9%) post-NACT. The findings of our study correlated with those of the studies done by Hurley et al. [14], Krishna et al. [15], Kunnuru et al. [12], and Blumencranz et al. [16]. In their study, Hurley et al. found a significant reduction in TN staging post-NACT [14]. Krishna et al. noted a significant reduction in tumor size after NACT [15]. Kunnuru et al. [12] noted that NACT led to a significant downstaging of the tumor and axillary metastasis in patients with LABC, and Blumencranz et al. [16] found cPR in the lymph nodes post-NACT. Thus, it can be inferred that NACT has a definitive role in downstaging the breast cancer tumor and lymph nodes.

In our study, 98.2% of the patients had IDC, while ILC was found in only one patient. Among the patients having IDC, cPR was noted in 21.4% of the patients post-NACT, whereas patients having ILC did not respond to NACT. Thus, as per the HPE findings, a significant improvement in the tumor status was noted post-NACT in patients having IDC in our study. These findings of our study were comparable to the findings of the studies done by Agarwal et al. [17], Mermut et al. [18], and Beresford et al. [19]. Agarwal et al., in their study, noted cPR in 16.1% of the patients post-NACT [17]. Mermut et al. [18] found IDC as the most commonly occurring histological subtype with cPR in 28% of the patients post-NACT, and Beresford et al. [19] noted cPR in 21.5% after NACT. Alawad did a study on 98 patients with LABC and treated them with NACT [20]. The authors noted that 76 (77.6%) patients had IDC, 15 (15.3%) had ILC, and seven (7.1%) had other subtypes. Out of these 76 patients with IDC, 72 patients showed either a complete or partial response to NACT. Only one patient with ILC (1%) had a complete clinical response to NACT. These findings suggest that the histological subtype in breast carcinoma plays a crucial role in determining the degree of clinical and pathological response to NACT. Moreover, it also raises the question of whether NACT should be instituted in patients with ILC.

In our study, as compared to pre-NACT, a significant downgrading of the breast tumor was noted post-NACT, with cPR being in 12 (21.4%) patients. This finding of our study was similar to the finding of a study done by Beresford et al. [19] and Gafoor et al. [21], in which post-NACT, the authors noted cPR in 21.5% and 21.3% of patients, respectively, suggesting that NACT lowers the clinical stage and grade of the LABC and is associated with the achievement of cPR, leading to an overall better prognosis.

In our study, out of 56 patients, in 44 patients, no change was found in the ER, PR, and HER2-neu receptor status pre- and post-NACT. The information regarding the transformation of receptor status post-NACT could not be evaluated in the remaining 12 patients due to the cPR of the tumor. This finding of our study was consistent with the finding of Faneyte et al., in which the authors noted that the change in receptor status pre- and post-NACT was statistically insignificant [22].

In our study, among various molecular subtypes, patients belonging to TNBC had the highest cPR rate post-NACT, comprising 12.5%, followed by luminal B (5.4%) and HER2-neu-positive subtype (3.5%), whereas no change was noted in the patients belonging to the luminal A subtype. This finding of our study was similar to the finding of Kim et al., in which the authors noted that post-NACT, cPR rate was maximum in TNBC (21.1%), followed by HER2-neu-positive (10.5%), luminal B (5.0%), and luminal A (3.9%) subtypes [23]. Thus, based on the above findings, it can be inferred that among various subtypes, TNBC is most sensitive to NACT.

Strengths

The major strengths of our study were the use of a comprehensive chemotherapy protocol, a clear research focus, and an in-depth analysis of findings.

Limitations

There were a few limitations to our study. Firstly, our study was exclusively based on a uniform patient population comprising female breast cancer patients diagnosed with LABC and who received NACT. So, the findings of this study may not apply to male breast cancer patients or other patient populations having a more generalized breast cancer disease. Secondly, our study was conducted at a single tertiary care center with a limited sample size. The results could have been different in the case of a multicentric study with a larger sample size. Thirdly, a long-term follow-up of the patients was not done in our study, which makes it difficult to confirm the lasting effect of NACT.

## Conclusions

In our study, a significant downstaging and downgrading of the tumor and downstaging of the lymph node status were observed following NACT in female patients diagnosed with LABC. Regarding the response of different molecular subtypes to NACT, the cPR rate was maximum in the TNBC subtype.

Thus, we conclude that in our study, NACT has a definitive role in female patients diagnosed with LABC as far as the clinical and pathological response of the tumor is concerned. Also, the TNBC subtype responded maximally to NACT in patients diagnosed with LABC and carried a better prognosis. The lack of long-term follow-up makes it difficult to confirm the lasting impact of NACT. Prospective, multicentric studies with a larger sample size are needed to validate these findings.

## References

[1] Arnold M, Morgan E, Rumgay H (2022). Current and future burden of breast cancer: global statistics for 2020 and 2040. Breast.

[2] Anderson BO, Ilbawi AM, Fidarova E, Weiderpass E, Stevens L, Abdel-Wahab M, Mikkelsen B (2021). The Global Breast Cancer Initiative: a strategic collaboration to strengthen health care for non-communicable diseases. Lancet Oncol.

[3] Koscielny S, Tubiana M, Lê MG, Valleron AJ, Mouriesse H, Contesso G, Sarrazin D (1984). Breast cancer: relationship between the size of the primary tumour and the probability of metastatic dissemination. Br J Cancer.

[4] Asaoka M, Gandhi S, Ishikawa T, Takabe K (2020). Neoadjuvant chemotherapy for breast cancer: past, present, and future. Breast Cancer (Auckl).

[5] Waljee JF, Newman LA (2007). Neoadjuvant systemic therapy and the surgical management of breast cancer. Surg Clin North Am.

[6] Carter CL, Allen C, Henson DE (1989). Relation of tumor size, lymph node status, and survival in 24,740 breast cancer cases. Cancer.

[7] Valagussa P, Zambetti M, Bignami P (1983). T3b-T4 breast cancer: factors affecting results in combined modality treatments. Clin Exp Metastasis.

[8] Aebi S, Karlsson P, Wapnir IL (2022). Locally advanced breast cancer. Breast.

[9] Teichgraeber DC, Guirguis MS, Whitman GJ (2021). Breast cancer staging: updates in the AJCC cancer staging manual, 8th edition, and current challenges for radiologists, from the AJR special series on cancer staging. AJR Am J Roentgenol.

[10] Bloom HJ, Richardson WW (1957). Histological grading and prognosis in breast cancer; a study of 1409 cases of which 359 have been followed for 15 years. Br J Cancer.

[11] Eisenhauer EA, Therasse P, Bogaerts J (2009). New response evaluation criteria in solid tumours: revised RECIST guideline (version 1.1). Eur J Cancer.

[12] Kunnuru SK, Thiyagarajan M, Martin Daniel J, Singh K B (2020). A study on clinical and pathological responses to neoadjuvant chemotherapy in breast carcinoma. Breast Cancer (Dove Med Press).

[13] Nelson HD, Fu R, Cantor A, Pappas M, Daeges M, Humphrey L (2016). Effectiveness of breast cancer screening: systematic review and meta-analysis to update the 2009 U.S. Preventive Services Task Force recommendation. Ann Intern Med.

[14] Hurley J, Reis IM, Rodgers SE (2013). The use of neoadjuvant platinum-based chemotherapy in locally advanced breast cancer that is triple negative: retrospective analysis of 144 patients. Breast Cancer Res Treat.

[15] Krishna NM, Mani CM, Veera AC, Chandra TJ (2020). Evaluation of neo adjuvant chemotherapy response in patients with locally advanced breast cancer. Int J Clin Trials.

[16] Blumencranz P, Habibi M, Treece T (2019). Abstract PD8-04: neoadjuvant chemotherapy for breast cancer: nodal downstaging is highly correlated with pathological complete response. Cancer Res.

[17] Agarwal S, Pandey P, Ralli M, Chaturvedi V, Mittal K, Singh S (2019). Assessment of pathological response to neoadjuvant chemotherapy in patients with breast carcinoma using Sataloff system. Arch Oncol.

[18] Mermut O, Inanc B, Gursu RU, Arslan E, Trabulus DC, Havare SB, Ulusan MB (2021). Factors affecting pathological complete response after neoadjuvant chemotherapy in breast cancer: a single-center experience. Rev Assoc Med Bras (1992).

[19] Beresford MJ, Stott D, Makris A (2008). Assessment of clinical response after two cycles of primary chemotherapy in breast cancer. Breast Cancer Res Treat.

[20] Alawad AA (2014). Evaluation of clinical and pathological response after two cycles of neoadjuvant chemotherapy on Sudanese patients with locally advanced breast cancer. Ethiop J Health Sci.

[21] Gafoor AF, Balakrishnan P, Krishna KJ, Arjunan A (2022). Evaluation of clinical and pathological response in breast cancer following neoadjuvant chemotherapy- a single institution experience. J Clin Diagn Res.

[22] Faneyte IF, Schrama JG, Peterse JL, Remijnse PL, Rodenhuis S, van de Vijver MJ (2003). Breast cancer response to neoadjuvant chemotherapy: predictive markers and relation with outcome. Br J Cancer.

[23] Kim SI, Sohn J, Koo JS, Park SH, Park HS, Park BW (2010). Molecular subtypes and tumor response to neoadjuvant chemotherapy in patients with locally advanced breast cancer. Oncology.

